# 8-Hy­droxy­quinolinium tri­chlorido­(pyridine-2,6-di­carb­oxy­lic acid-κ^3^*O*,*N*,*O*′)copper(II) dihydrate

**DOI:** 10.1107/S2056989024009186

**Published:** 2024-09-24

**Authors:** Yusufjon Eshkobilovich Nazarov, Khayit Khudainazarovich Turaev, Jabbor Ruziboevich Suyunov, Bekmurod Khurramovich Alimnazarov, Abdusamat Abdujabborovich Rasulov, Bakhtiyar Tulyaganovich Ibragimov, Jamshid Mengnorovich Ashurov

**Affiliations:** a Termez State University, Barkamol Avlod Street 43, Termez City, Uzbekistan; b Institute of Bioorganic Chemistry, Academy of Sciences of Uzbekistan, 100125, M. Ulugbek Str. 83, Tashkent, 700125, Uzbekistan; Illinois State University, USA

**Keywords:** pyridine-2,6-di­carb­oxy­lic acid, 8-hy­droxy­quinoline, crystal structure, hydrogen bonds, Hirshfeld surface analysis

## Abstract

The title compound, (C_9_H_8_NO)[CuCl_3_(C_7_H_5_NO_4_)]·2H_2_O, was prepared by reacting Cu^II^ acetate dihydrate, solid 8-hy­droxy­quinoline (8-HQ), and solid pyridine-2,6-di­carb­oxy­lic acid (H_2_pydc), in a 1:1:1 molar ratio, in an aqueous solution of dilute hydro­chloric acid. The Cu^II^ atom exhibits a distorted CuO_2_NCl_3_ octa­hedral geometry, coordinating two oxygen atoms and one nitro­gen atom from the tridentate H_2_pydc ligand and three chloride atoms; the nitro­gen atom and one chloride atom occupy the axial positions with Cu—N and Cu—Cl bond lengths of 2.011 (2) Å and 2.2067 (9) Å, respectively. In the equatorial plane, the oxygen and chloride atoms are arranged in a *cis* configuration, with Cu—O bond lengths of 2.366 (2) and 2.424 (2) Å, and Cu—Cl bond lengths of 2.4190 (10) and 2.3688 (11) Å. The asymmetric unit contains 8-HQ^+^ as a counter-ion and two uncoordinated water mol­ecules. The crystal structure features strong O—H⋯O and O—H⋯Cl hydrogen bonds as well as weak inter­actions including C—H⋯O, C—H⋯Cl, Cu—Cl⋯π, and π–π, which result in a three-dimensional network. A Hirshfeld surface analysis indicates that the most important contributions to the crystal packing involving the main residues are from H⋯Cl/Cl⋯H inter­actions, contributing 40.3% for the anion. Weak H⋯H contacts contribute 13.2% for the cation and 28.6% for the anion.

## Abstract 2

The title compound, (C_9_H_8_NO)[CuCl_3_(C_7_H_5_NO_4_)]·2H_2_O, was synthesized by reacting Cu^II^ acetate, 8-hy­droxy­quinoline, and pyridine-2,6-di­carb­oxy­lic acid in dilute hydro­chloric acid in a 1:1:1 molar ratio. The Cu^II^ atom exhibits a distorted octa­hedral geometry coordinated by the H_2_pydc ligand and chloride atoms. In the crystal structure, various hydrogen bonds and weak inter­actions lead to the formation of a three-dimensional network.

## 1. Chemical context

8-Hy­droxy­quinoline (8HQ, C_9_H_7_NO), also known as oxine, is a versatile bidentate chelating agent forming species such as H_2_*L*^+^, H*L*, and *L*^−^. With p*K*a values of 10.8 and 4.9 for the nitro­gen and phenol groups, respectively, it effectively forms supra­molecular structures through hydrogen bonding (Smith *et al.*, 2003 ▸). 8HQ is extensively utilized in analytical chemistry for metal-ion qu­anti­fication because of the insolubility of its complexes in water (Albrecht *et al.*, 2008 ▸). Tris(8-hy­droxy­quinolinato)aluminum is crucial in OLEDs (Cölle *et al.*, 2002 ▸; Katakura & Koide, 2006 ▸), and its luminescence properties are enhanced by ring substituents (Montes *et al.*, 2006 ▸). Its metal binding induces fluorescence changes, useful in developing sensitive chemosensors for detecting metal ions like zinc, cadmium, lead, and mercury (Moon *et al.*, 2004 ▸; Zhang *et al.*, 2005 ▸; Farruggia *et al.*, 2006 ▸; Mei *et al.*, 2006 ▸). 8HQ derivatives enhance adsorbents for heavy-metal removal from solutions (Kosa *et al.*, 2012 ▸) and serve as corrosion inhibitors in acidic media (Rbaa *et al.*, 2018 ▸).

Quinoline derivatives, including 8HQ, exhibit a broad spectrum of biological activities in medicinal chemistry (Song *et al.*, 2014 ▸; Cherdtrakulkiat *et al.*, 2016 ▸), showing anti­microbial, anti­oxidant, anti­cancer, anti-inflammatory, anti­neurodegenerative, anti­malarial, and anti­tuberculotic activities (Song *et al.*, 2015 ▸; Cherdtrakulkiat *et al.*, 2016 ▸; Dixit *et al.*, 2010 ▸).

Copper(II) complexes of 8HQ derivatives show potential in treating Alzheimer’s disease (Qin *et al.*, 2015 ▸), while their anti­microbial properties are attributed to metal ion chelation (Dixit *et al.*, 2010 ▸; Yin *et al.*, 2020 ▸). Pyridine-2,6-di­carb­oxy­lic acid (H_2_pydc) has a p*K*a of 2.16 at 25°C. This ligand is notable for forming stable chelates with various metal ions, due to its two carboxyl groups arranged at 120°. It supports multiple coordination geometries, including bidentate, tridentate, meridional, and bridging modes (Yang *et al.*, 2015 ▸; Ye *et al.*, 2005 ▸). Its flexibility allows for the creation of both discrete and polymeric metal complexes (Aghabozorg *et al.*, 2008 ▸). H_2_pydc is essential for constructing some metal–organic frameworks (MOFs) for applications in adsorption, catalysis, and photoluminescence (Cui *et al.*, 2012 ▸; Tanner *et al.*, 2010 ▸). These complexes also exhibit significant anti­microbial and anti­cancer activities (Li *et al.*, 2014 ▸; Shi *et al.*, 2009 ▸). Additionally, many co-crystals and proton-transfer compounds involving H_2_pydc have been studied (Zhang *et al.*, 2015 ▸). In our previous work (Naza­rov *et al.*, 2024 ▸), we reported on the organic salt of bis­(8-hy­droxy­quinolinium) naphthalene-1,5-di­sulfonate tetra­hydrate. In this paper, we focus on the synthesis and structural characterization of the salt formed from 8-hy­droxy­quinoline and pyridine-2,6-di­carb­oxy­lic acid in dilute hydro­chloric acid.
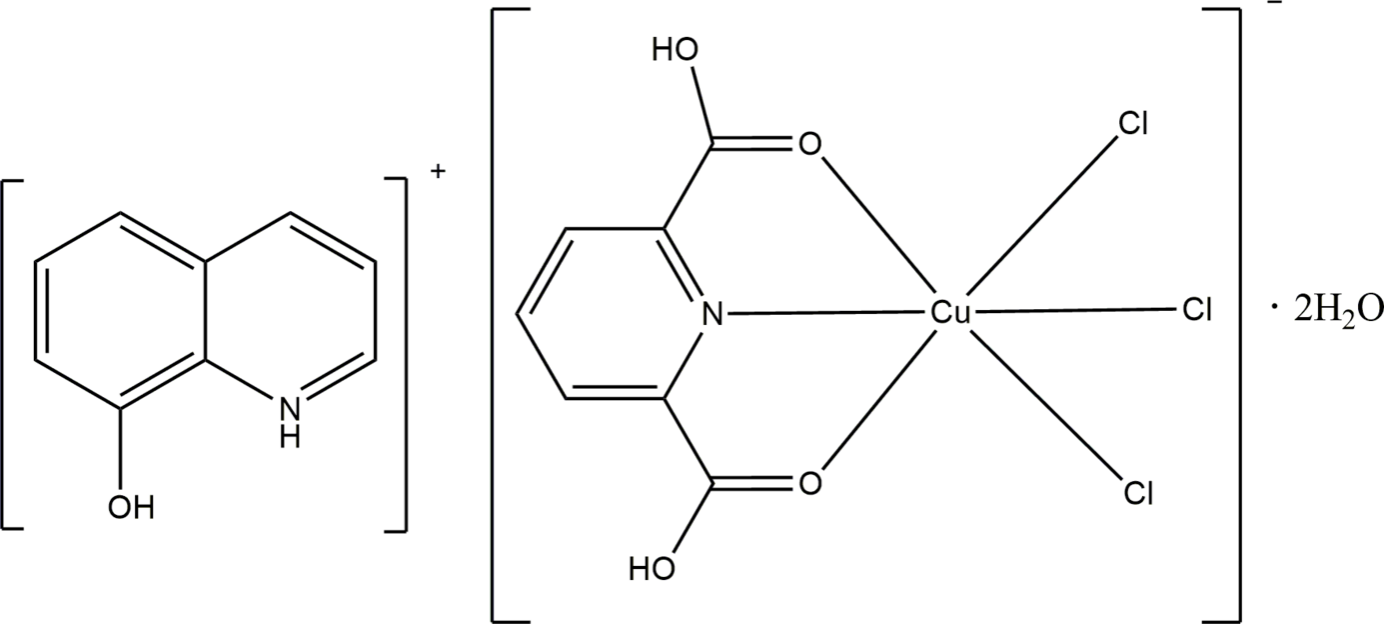


## 2. Structural commentary

**Figure 1:**
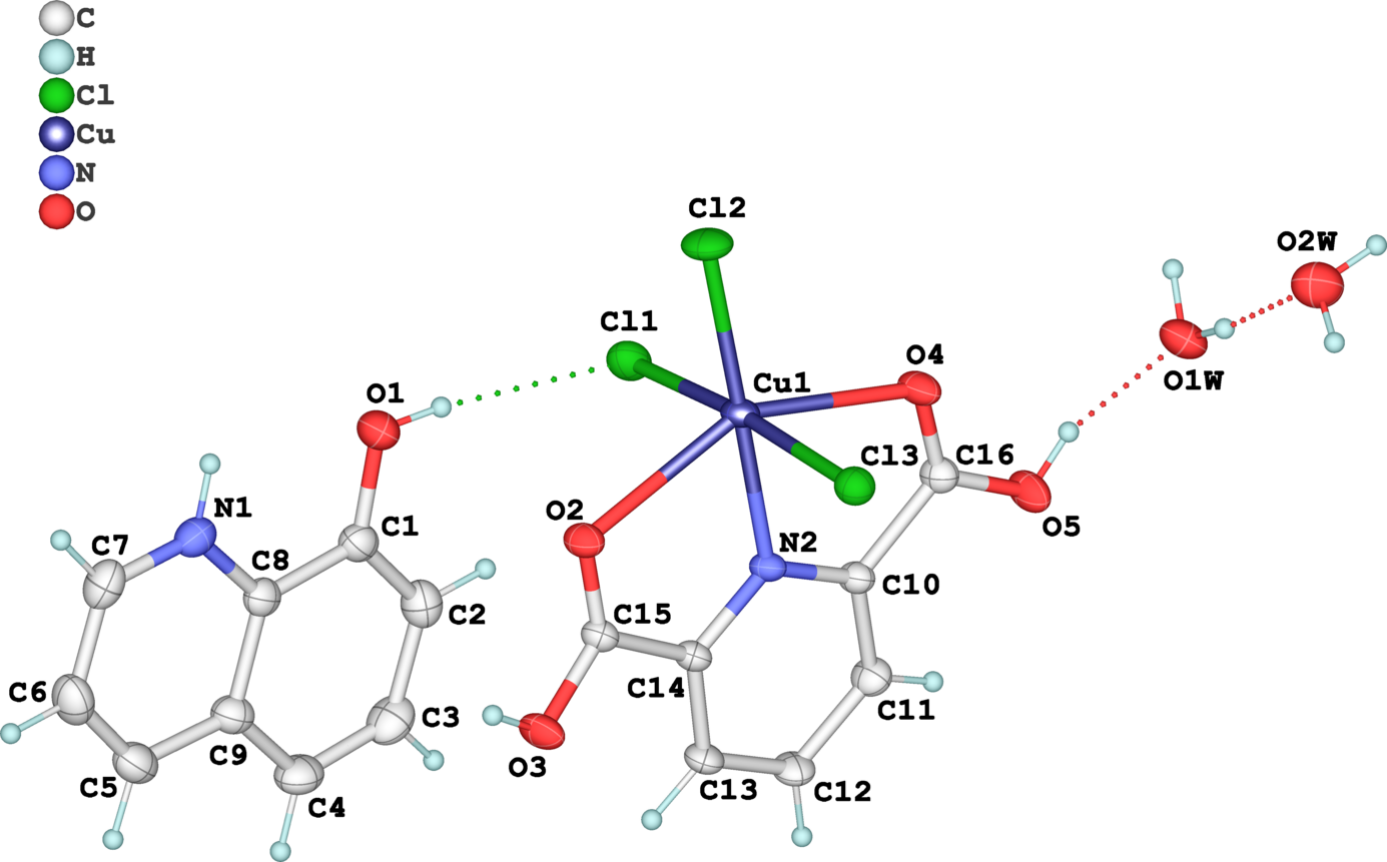
The structures of the mol­ecular entities in the title salt, showing the atom-labeling scheme and displacement ellipsoids drawn at the 50% probability level. H atoms are shown as spheres of arbitrary radius and hydrogen bonds are shown as dashed lines.

**Figure 2:**
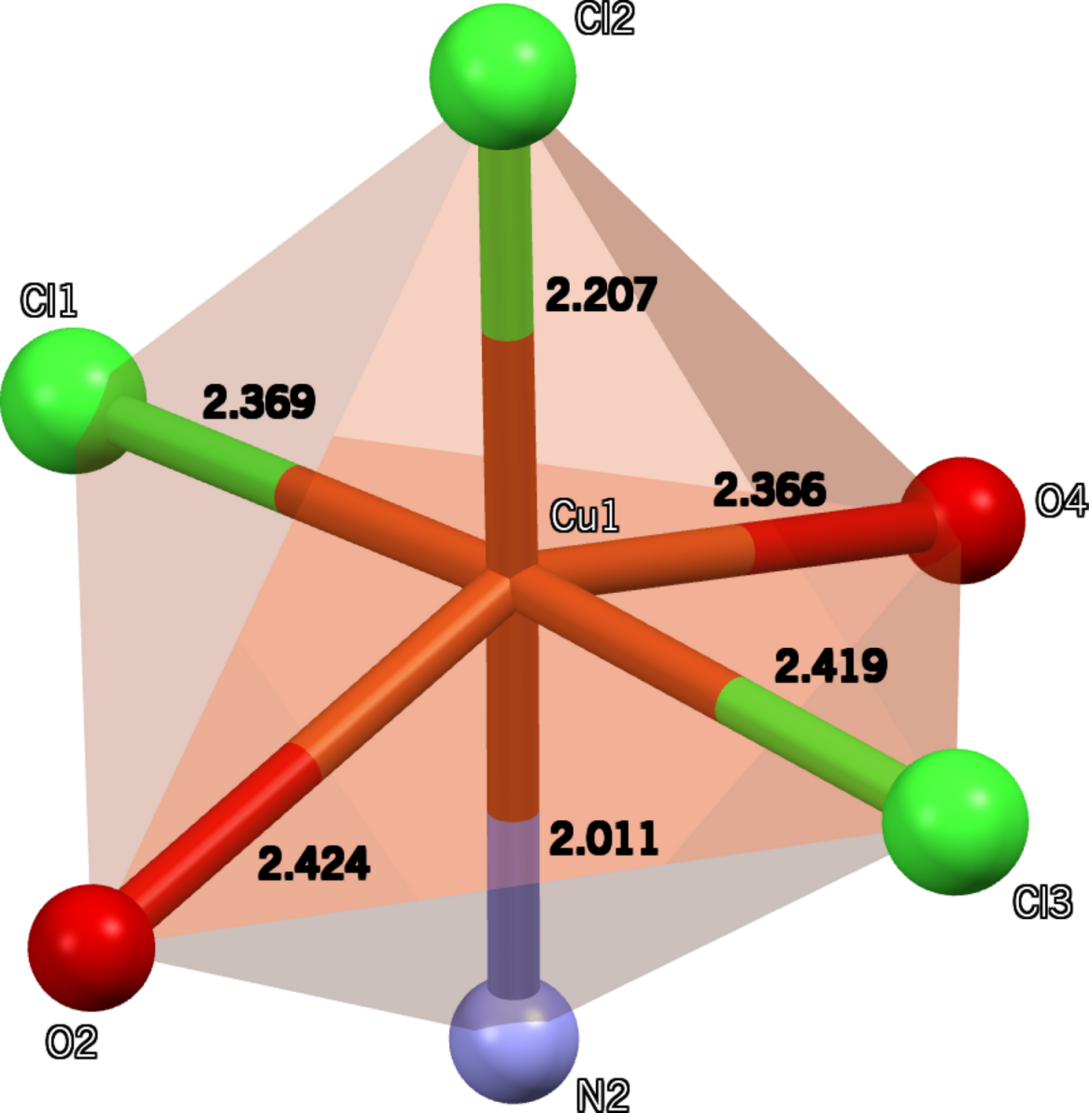
Coordination polyhedron around the copper cation, with other atoms omitted for clarity.

The title hydrated mol­ecular salt consists of a [Cu(H_2_pydc)Cl_3_]^−^ anion, 8HQ^+^ cation and two uncoordinated water mol­ecules (Fig. 1 ▸). The Cu^II^ atom exhibits a distorted CuO_2_NCl_3_ octa­hedral geometry (Fig. 2 ▸). It coordinates two oxygen atoms and one nitro­gen atom from the tridentate H_2_pydc ligand, along with three chloride ions. The Cu—N bond length is 2.011 (2) Å, while the Cu—O bond lengths are 2.366 (2) and 2.424 (2) Å. The Cu—Cl bond lengths are 2.2067 (9), 2.3688 (11) and 2.4190 (10) Å. The *cis* angles range from 74.48 (9) to 105.45 (6)°, and the *trans* angles range from 149.30 (8) to 174.14 (3)°. The pyridine ring of the H_2_pydc mol­ecule exhibits a planar geometry, with the maximum deviation of a ring atom from the least-squares plane being 0.007 Å. The carboxyl­ate groups attached to the pyridine ring form different dihedral angles of 11.094 (10) and 6.513 (1)° with the pyridine plane. This difference possibly results from the different bonding modes and inter­molecular hydrogen bonds with the O—H group. The 8-HQ unit is protonated, and the hy­droxy­quinoline cation fragment is also planar, with a maximum deviation of 0.0162 (14) Å. This fragment is coplanar with the plane of the H_2_pydc mol­ecule.

## 3. Supra­molecular features and Hirshfeld surface analysis

**Figure 3:**
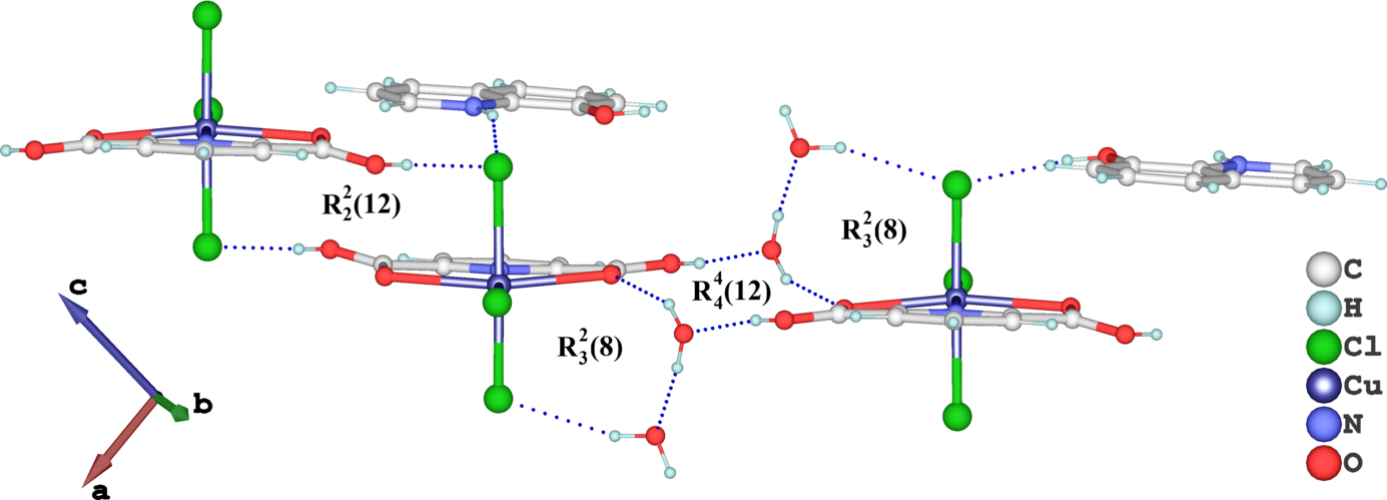
The formation of O—H⋯O, O—H⋯Cl and N—H⋯Cl hydrogen bonds (dashed lines) in the crystal structure, leading to 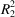
(12), 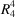
(12) and 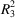
(8) graph-set motifs.

**Figure 4:**
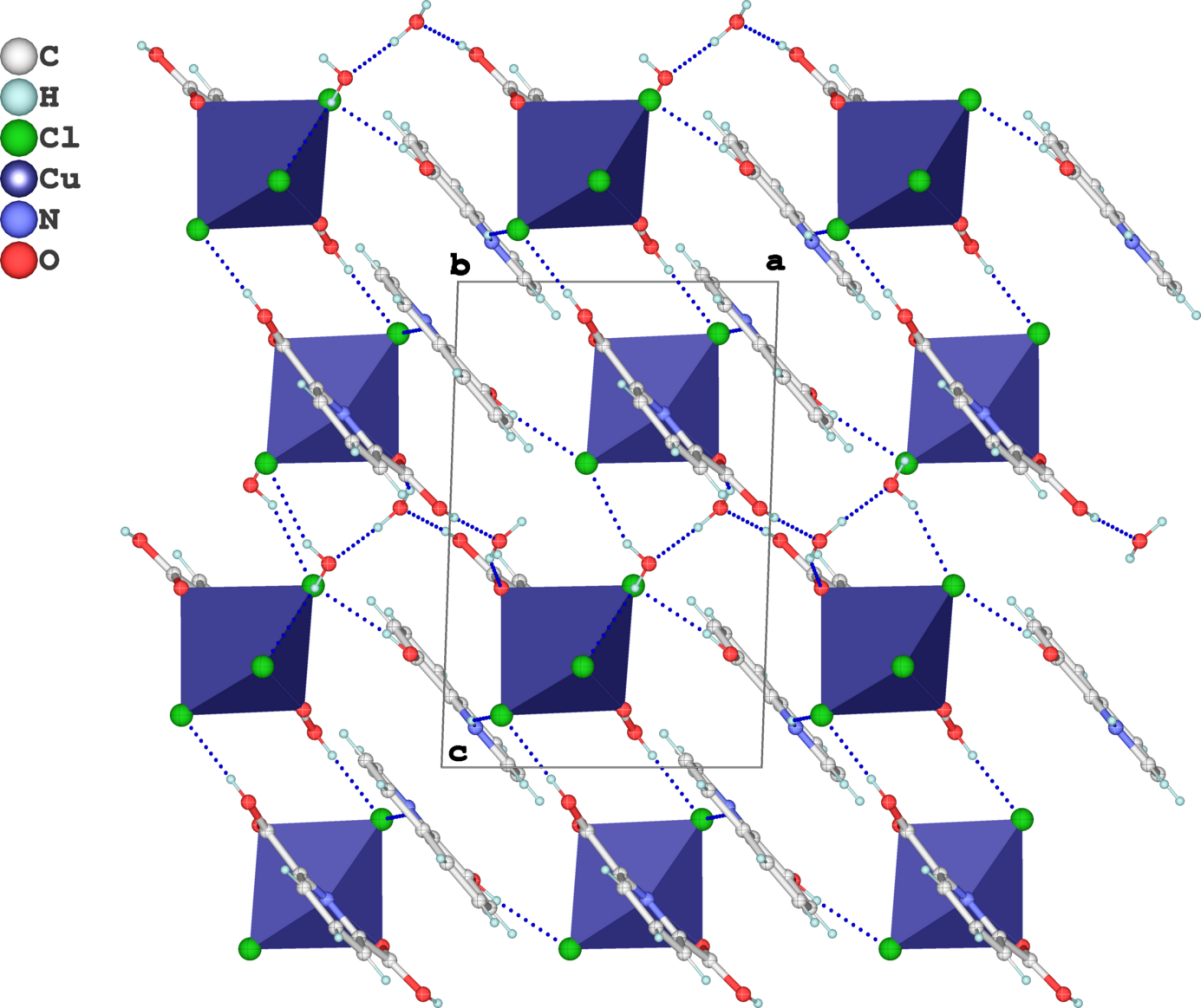
The crystal packing of the title salt in a view along [010]. O—H⋯O, O—H⋯Cl and N—H⋯Cl hydrogen bonds are shown as dashed blue lines.

**Figure 5:**
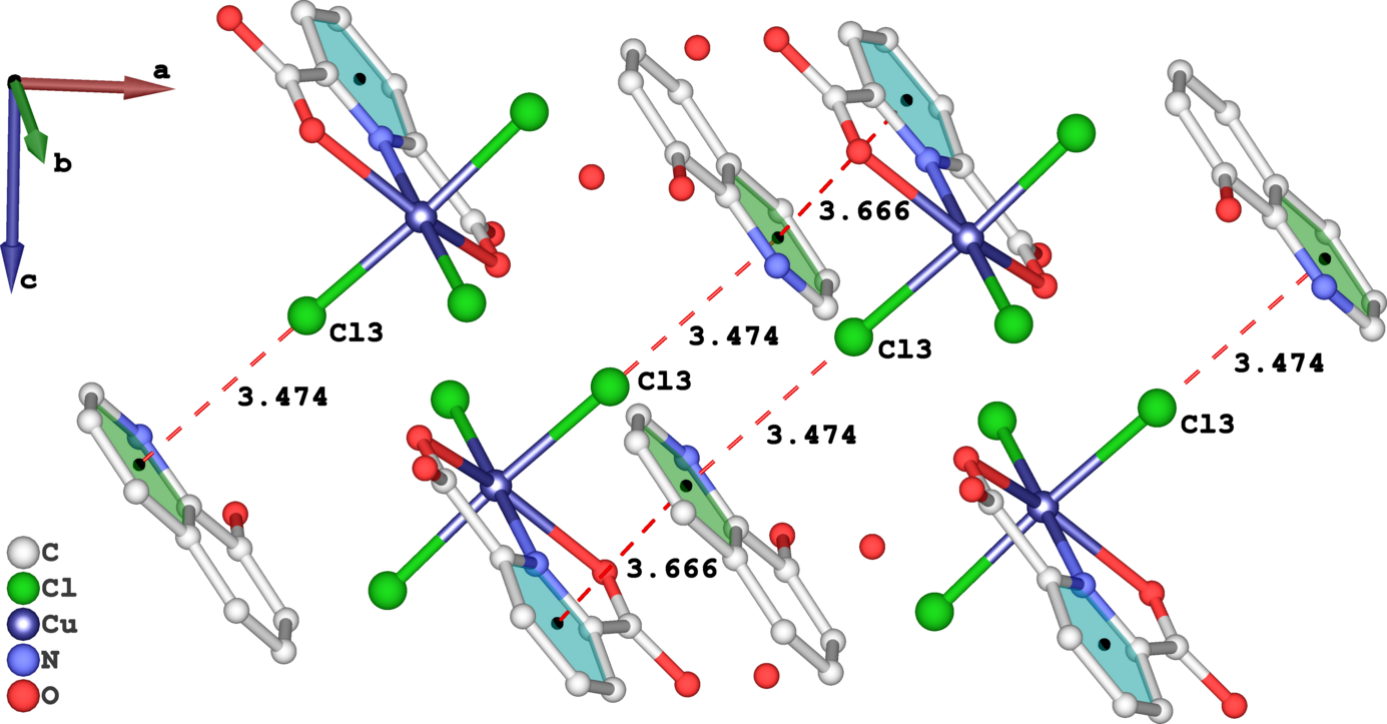
A fragment of the packing of the title compound showing Cu—Cl⋯π and π-π- inter­actions between the pyridine rings of H_2_pydc and the 8HQ^+^ cation.

In the crystal, the 8HQ^+^ cation, the [Cu(H_2_pydc)Cl_3_]^−^ anion, and the water mol­ecules are connected *via* strong O—H⋯O and O—H⋯Cl hydrogen bonds (Table 1 ▸) with graph-set motifs of 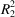
(12), 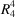
(12) and 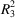
(8) (Fig. 3 ▸), which link the components into chains extending along [100] and [0
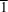
1], forming a two-dimensional network lying in the (011) plane (Fig. 4 ▸). All chlorine atoms in the anion participate in hydrogen bonding. As depicted in Fig. 5 ▸, the Cl3 atom exhibits Cu—Cl⋯π inter­actions with the pyridine ring of 8HQ [Cl⋯*Cg*2^iii^ = 3.4736 (17) Å; Cu—Cl⋯*Cg*2^iii^ = 167.79 (4)°; *Cg*2 is the centroid of the 8HQ pyridine ring; symmetry code: (iii) 1 − *x*, 1 − *y*, −*z*]. There is also an extensive π–π inter­action between the rings of H2pydc and 8HQ^+^ cation fragments, with centroid–centroid distances for *Cg*1⋯*Cg*2^iv^ of 3.666 (2) Å, where *Cg*1 is the centroid of the N2/C10–C14 ring [symmetry code: (iv) 1 + *x*, *y*, *z*].

**Figure 6:**
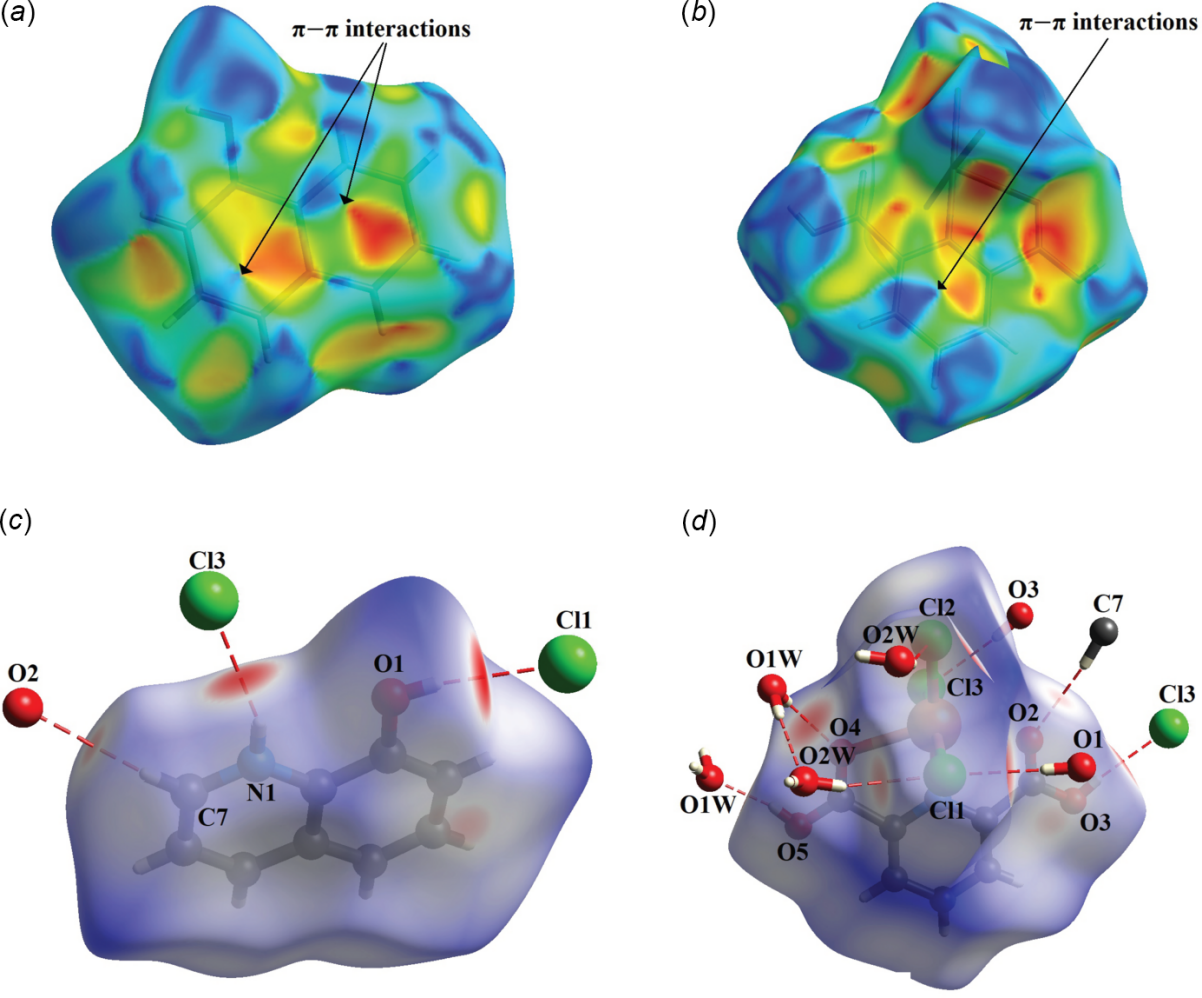
Hirshfeld surfaces mapped over *d*_norm_ and shape index for (*a*), (*c*) the 8HQ^+^ cation and (*b*), (*d*) the [Cu(H_2_pydc)Cl_3_]^−^ anion, respectively.

**Figure 7:**
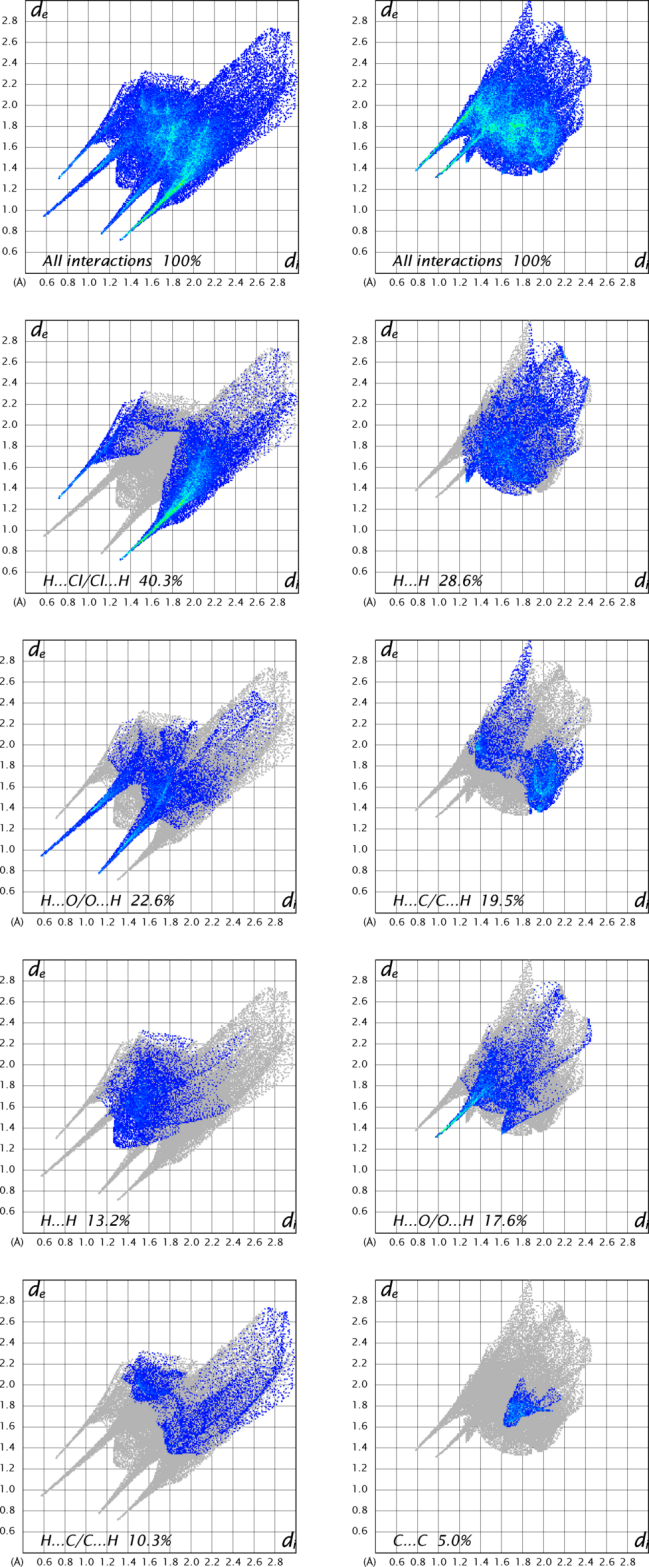
Two-dimensional fingerprint plots for the [Cu(H_2_pydc)Cl_3_]^−^ anion (left) and the 8HQ^+^ cation (right), showing all contributions and contributions between specific inter­acting atom pairs.

In the crystal packing, a wide range of non-covalent inter­actions, consisting of hydrogen bonding, Cu—Cl⋯π, and π–π inter­actions, play an important role in the cohesion of the three-dimensional supra­molecular network. In order to visualize the inter­molecular inter­actions in the structure of the title compound, a Hirshfeld surface (HS) analysis was carried out (Spackman & Jayatilaka, 2009 ▸) and the associated two-dimensional fingerprint plots (McKinnon *et al.*, 2007 ▸) were generated using *CrystalExplorer 21.5* (Spackman *et al.*, 2021 ▸). The presence of strong inter­actions on the Hirshfeld surface is indicated by red spots, while the blue areas indicate weak inter­actions, as shown in Fig. 6 ▸. The two-dimensional fingerprint plot for all inter­actions and those delineated into individual inter­actions, together with their relative contributions, are shown in Fig. 7 ▸. The Hirshfeld surface analysis indicates that the most important contributions to the crystal packing involving the main residues are from H⋯Cl/Cl⋯H inter­actions, contributing 40.3% for the anion. Weak H⋯H contacts contribute 13.2% for the cation and 28.6% for the anion. O⋯H/O⋯H inter­actions contribute 22.6% for the cation and 17.6% for the anion, while H⋯C/C⋯H inter­actions contribute 19.5% for the cation and 10.3% for the anion. The Hirshfeld surface (HS) shape index is a tool used to visualize π–π stacking inter­actions, indicated by the presence of adjacent red and blue triangles. Fig. 6 ▸ clearly shows that π–π inter­actions are present in both the pyridine ring of the H_2_pydc mol­ecule and in both the pyridine and phenyl rings of the 8HQ^+^ cation.

## 4. Database survey

A search of the Cambridge Structural Database (CSD, version 5.45, updated November 2023; Groom *et al.*, 2016 ▸) revealed that the crystal structure of 8HQ has been determined; 27 reports are related to neutral structures. In addition, there are over 100 reports of organic salts and co-crystals and over 100 reports of metal complexes, among which 25 are chelates. In 150 cases, the nitro­gen atom of 8HQ is protonated. There are seven cases where 8-hy­droxy­quinolinium and pyridine-2,6-di­carboxyl­ate are simultaneously present in the same compound. During the search, more than 2600 compounds of H_2_pydc and its deprotonated form were found. About 250 of them are organic salts and co-crystals, while the rest are metallocomplexes, more than 2200 of which are tridentately coordinated. Additionally, there are instances where H_2_pydc in its neutral form is tridentately coordinated to copper(II), as seen in the complexes LACGUT (Fainerman-Melnikova *et al.*, 2010 ▸) and QIDSAY (Prasad *et al.*, 2007 ▸).

## 5. Synthesis and crystallization

The title compound, (C_9_H_8_NO)[CuCl_3_(C_7_H_5_NO_4_)]·2H_2_O, was prepared by the reaction of Cu^II^ acetate dihydrate (0.2357 g, 1.083 mmol) in dilute hydro­chloric acid, 8-hy­droxy­quinoline (8-HQ) (0.1452 g, 0.9934 mmol), and pyridine-2,6-di­carb­oxy­lic acid (H_2_pydc) (0.1671 g, 1.000 mmol) in a 1:1:1 molar ratio in an aqueous solution. Good-quality single crystals were obtained by slow evaporation after four days (yield: 60%). Elemental analysis for C_16_H_17_Cl_3_CuN_2_O_7_ (519.20): calculated C: 37.01, H: 3.30, N:5.40%; found: C: 36.92, H: 3.28, N: 5.36%.

## 6. Refinement

Crystal data, data collection and structure refinement details are summarized in Table 2 ▸. C-bound H atoms were positioned geometrically and refined as riding with *U*_iso_(H) = 1.2*U*_eq_(C). The following restrains were used for N- and O-bound H atoms: N1—H1*A =* 0.86±0.01 Å, O1—H1 = O3—H3 = 0.82±0.01 Å, O5—H5 = 0.82±0.01 Å, O1*W*—H1*WB* = O1*W*—H1*WA* = 0.85±0.01 Å, O2*W*—H2*WB* = O2*W*—H2*WA* = 0.85±0.01 Å.

## Supplementary Material

Crystal structure: contains datablock(s) I. DOI: 10.1107/S2056989024009186/ej2008sup1.cif

Structure factors: contains datablock(s) I. DOI: 10.1107/S2056989024009186/ej2008Isup2.hkl

CCDC reference: 2307012

Additional supporting information:  crystallographic information; 3D view; checkCIF report

## Figures and Tables

**Table 1:** Hydrogen-bond geometry (Å, °)

*D*—H⋯*A*	*D*—H	H⋯*A*	*D*⋯*A*	*D*—H⋯*A*
O1—H1⋯Cl1	0.82 (4)	2.31 (4)	3.124 (3)	172 (3)
O1*W*—H1*WA*⋯O2*W*	0.85 (4)	1.86 (4)	2.697 (5)	172 (5)
N1—H1*A*⋯Cl3^i^	0.86 (2)	2.41 (3)	3.201 (3)	154 (5)
O1*W*—H1*WB*⋯O4^ii^	0.84 (4)	2.04 (3)	2.808 (4)	152 (4)
O3—H3⋯Cl3^iii^	0.82 (3)	2.20 (3)	3.015 (3)	173 (4)
O2*W*—H2*WA*⋯Cl2^iv^	0.86 (6)	2.53 (5)	3.361 (4)	163 (5)
O2*W*—H2*WB*⋯Cl1^ii^	0.85 (5)	2.50 (5)	3.317 (4)	160 (4)
O5—H5⋯O1*W*	0.82 (4)	1.69 (4)	2.477 (4)	162 (5)
C3—H3*A*⋯O2*W*^v^	0.93	2.56	3.473 (5)	168
C6—H6⋯Cl2^vi^	0.93	2.78	3.622 (4)	151
C7—H7⋯O2^vi^	0.93	2.44	3.356 (4)	169
C11—H11⋯O5^vii^	0.93	2.56	3.396 (5)	150
C12—H12⋯O1*W*^vii^	0.93	2.58	3.412 (5)	148

Symmetry codes: (i) 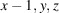; (ii) 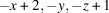; (iii) 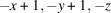; (iv) 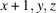; (v) 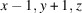; (vi) 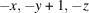; (vii) 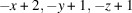.

**Table 2:** Experimental details

Crystal data	
Chemical formula	(C_9_H_8_NO)[CuCl_3_(C_7_H_5_NO_4_)]·2H_2_O
*M*_r_	519.20
Crystal system, space group	Triclinic, *P* 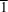
Temperature (K)	291
*a*, *b*, *c* (Å)	8.4699 (5), 9.7818 (5), 12.9026 (11)
α, β, γ (°)	77.238 (6), 89.207 (6), 78.038 (5)
*V* (Å^3^)	1019.37 (12)
*Z*	2
Radiation type	Cu *K*α
μ (mm^−1^)	5.52
Crystal size (mm)	0.26 × 0.24 × 0.18

Data collection	
Diffractometer	Xcalibur, Ruby
Absorption correction	Multi-scan (*CrysAlis PRO*; Rigaku OD, 2022 ▸)
*T*_min_, *T*_max_	0.819, 1.000
No. of measured, independent and observed [*I* > 2σ(*I*)] reflections	7074, 4134, 3092
*R*_int_	0.046
(sin θ/λ)_max_ (Å^−1^)	0.630

Refinement	
*R*[*F*^2^ > 2σ(*F*^2^)], *wR*(*F*^2^), *S*	0.042, 0.105, 1.01
No. of reflections	4134
No. of parameters	295
No. of restraints	8
H-atom treatment	H atoms treated by a mixture of independent and constrained refinement
Δρ_max_, Δρ_min_ (e Å^−3^)	0.39, −0.38

Computer programs: *CrysAlis PRO* (Rigaku OD, 2022 ▸), *SHELXT2018/2* (Sheldrick, 2015*a* ▸), *SHELXL2019/2* (Sheldrick, 2015*b* ▸), *OLEX2* (Dolomanov *et al.*, 2009 ▸), and *publCIF* (Westrip 2010 ▸).

## supplementary crystallographic information

### 8-Hydroxyquinolinium trichlorido(pyridine-2,6-dicarboxylic acid-κ3O,N,O')copper(II) dihydrate Crystal data

**Table:**

(C_9_H_8_NO)[CuCl_3_(C_7_H_5_NO_4_)]·2H_2_O	*Z* = 2
*M**_r_* = 519.20	*F*(000) = 526
Triclinic, *P*1	*D*_x_ = 1.692 Mg m^−^^3^
*a* = 8.4699 (5) Å	Cu *K*α radiation, λ = 1.54184 Å
*b* = 9.7818 (5) Å	Cell parameters from 2815 reflections
*c* = 12.9026 (11) Å	θ = 3.5–75.6°
α = 77.238 (6)°	µ = 5.52 mm^−^^1^
β = 89.207 (6)°	*T* = 291 K
γ = 78.038 (5)°	Block, light blue
*V* = 1019.37 (12) Å^3^	0.26 × 0.24 × 0.18 mm

### 8-Hydroxyquinolinium trichlorido(pyridine-2,6-dicarboxylic acid-κ3O,N,O')copper(II) dihydrate Data collection

**Table:**

Xcalibur, Ruby diffractometer	4134 independent reflections
Radiation source: fine-focus sealed X-ray tube, Enhance (Cu) X-ray Source	3092 reflections with *I* > 2σ(*I*)
Graphite monochromator	*R*_int_ = 0.046
Detector resolution: 10.2576 pixels mm^-1^	θ_max_ = 76.2°, θ_min_ = 3.5°
ω scans	*h* = −10→10
Absorption correction: multi-scan (CrysAlisPro; Rigaku OD, 2022)	*k* = −12→10
*T*_min_ = 0.819, *T*_max_ = 1.000	*l* = −15→16
7074 measured reflections	

### 8-Hydroxyquinolinium trichlorido(pyridine-2,6-dicarboxylic acid-κ3O,N,O')copper(II) dihydrate Refinement

**Table:**

Refinement on *F*^2^	Hydrogen site location: mixed
Least-squares matrix: full	H atoms treated by a mixture of independent and constrained refinement
*R*[*F*^2^ > 2σ(*F*^2^)] = 0.042	*w* = 1/[σ^2^(*F*_o_^2^) + (0.0387*P*)^2^] where *P* = (*F*_o_^2^ + 2*F*_c_^2^)/3
*wR*(*F*^2^) = 0.105	(Δ/σ)_max_ = 0.001
*S* = 1.01	Δρ_max_ = 0.39 e Å^−^^3^
4134 reflections	Δρ_min_ = −0.37 e Å^−^^3^
295 parameters	Extinction correction: *SHELXL2019/2* (Sheldrick, 2015b), Fc^*^=kFc[1+0.001xFc^2^λ^3^/sin(2θ)]^-1/4^
8 restraints	Extinction coefficient: 0.0043 (3)
Primary atom site location: iterative	

### 8-Hydroxyquinolinium trichlorido(pyridine-2,6-dicarboxylic acid-κ3O,N,O')copper(II) dihydrate Special details

**Table:**

Geometry. All esds (except the esd in the dihedral angle between two l.s. planes) are estimated using the full covariance matrix. The cell esds are taken into account individually in the estimation of esds in distances, angles and torsion angles; correlations between esds in cell parameters are only used when they are defined by crystal symmetry. An approximate (isotropic) treatment of cell esds is used for estimating esds involving l.s. planes.

### 8-Hydroxyquinolinium trichlorido(pyridine-2,6-dicarboxylic acid-κ3O,N,O')copper(II) dihydrate Fractional atomic coordinates and isotropic or equivalent isotropic displacement parameters (Å^2^)

**Table:**

	*x*	*y*	*z*	*U*_iso_*/*U*_eq_	
Cu1	0.61613 (6)	0.28127 (4)	0.24128 (4)	0.02919 (15)	
Cl1	0.42036 (11)	0.28777 (9)	0.37398 (7)	0.0420 (2)	
Cl2	0.56984 (12)	0.08134 (9)	0.20741 (8)	0.0451 (2)	
Cl3	0.81820 (10)	0.29892 (8)	0.10741 (6)	0.03518 (19)	
O2	0.4368 (3)	0.4718 (2)	0.11873 (18)	0.0346 (5)	
O3	0.4004 (3)	0.7115 (2)	0.0718 (2)	0.0405 (6)	
H3	0.344 (4)	0.701 (4)	0.024 (2)	0.041 (11)*	
O4	0.8324 (3)	0.2084 (2)	0.36886 (19)	0.0385 (6)	
O5	0.9662 (3)	0.3119 (3)	0.4671 (2)	0.0439 (6)	
H5	1.015 (7)	0.229 (2)	0.486 (5)	0.11 (2)*	
N2	0.6552 (3)	0.4647 (3)	0.27169 (19)	0.0261 (5)	
C10	0.7590 (4)	0.4598 (3)	0.3508 (2)	0.0282 (6)	
C11	0.7769 (4)	0.5813 (4)	0.3825 (3)	0.0351 (7)	
H11	0.848641	0.575175	0.438104	0.042*	
C12	0.6865 (4)	0.7131 (3)	0.3304 (3)	0.0358 (7)	
H12	0.695999	0.796604	0.350676	0.043*	
C13	0.5824 (4)	0.7179 (3)	0.2480 (3)	0.0329 (7)	
H13	0.522015	0.805217	0.210987	0.039*	
C14	0.5686 (4)	0.5918 (3)	0.2209 (2)	0.0273 (6)	
C15	0.4607 (4)	0.5844 (3)	0.1314 (2)	0.0289 (6)	
C16	0.8575 (4)	0.3121 (3)	0.3975 (2)	0.0315 (7)	
O1	0.1093 (3)	0.4401 (3)	0.2333 (2)	0.0463 (6)	
H1	0.186 (4)	0.396 (4)	0.274 (3)	0.063 (15)*	
N1	−0.1016 (4)	0.6102 (3)	0.0823 (2)	0.0376 (6)	
H1A	−0.089 (6)	0.5181 (12)	0.096 (4)	0.071 (15)*	
C1	0.0898 (4)	0.5833 (4)	0.2254 (3)	0.0367 (8)	
C2	0.1642 (4)	0.6467 (4)	0.2893 (3)	0.0444 (9)	
H2	0.237306	0.590373	0.342684	0.053*	
C3	0.1308 (5)	0.7977 (4)	0.2747 (3)	0.0498 (10)	
H3A	0.181465	0.839138	0.319434	0.060*	
C4	0.0262 (5)	0.8833 (4)	0.1966 (3)	0.0480 (9)	
H4	0.006611	0.982334	0.187777	0.058*	
C5	−0.1643 (5)	0.9014 (4)	0.0476 (3)	0.0460 (9)	
H5A	−0.188277	1.000856	0.035653	0.055*	
C6	−0.2383 (5)	0.8358 (5)	−0.0143 (3)	0.0516 (10)	
H6	−0.309582	0.889937	−0.069511	0.062*	
C7	−0.2064 (4)	0.6876 (4)	0.0058 (3)	0.0457 (9)	
H7	−0.258996	0.641940	−0.034829	0.055*	
C8	−0.0213 (4)	0.6706 (4)	0.1456 (3)	0.0338 (7)	
C9	−0.0523 (4)	0.8220 (4)	0.1293 (3)	0.0386 (8)	
O1W	1.1567 (4)	0.0804 (3)	0.5343 (3)	0.0505 (7)	
H1WA	1.224 (5)	0.043 (6)	0.494 (3)	0.09 (2)*	
H1WB	1.129 (6)	0.006 (3)	0.570 (4)	0.09 (2)*	
O2W	1.3740 (4)	−0.0607 (3)	0.4191 (3)	0.0569 (7)	
H2WA	1.414 (7)	−0.007 (6)	0.369 (4)	0.12 (2)*	
H2WB	1.444 (6)	−0.124 (5)	0.460 (4)	0.12 (3)*	

### 8-Hydroxyquinolinium trichlorido(pyridine-2,6-dicarboxylic acid-κ3O,N,O')copper(II) dihydrate Atomic displacement parameters (Å^2^)

**Table:**

	*U*^11^	*U*^22^	*U*^33^	*U*^12^	*U*^13^	*U*^23^
Cu1	0.0366 (3)	0.0193 (2)	0.0320 (3)	−0.00748 (18)	−0.00345 (19)	−0.00455 (17)
Cl1	0.0436 (5)	0.0361 (4)	0.0420 (5)	−0.0075 (4)	0.0053 (4)	−0.0005 (3)
Cl2	0.0609 (6)	0.0261 (4)	0.0537 (5)	−0.0171 (4)	0.0002 (4)	−0.0127 (3)
Cl3	0.0375 (4)	0.0329 (4)	0.0357 (4)	−0.0071 (3)	0.0002 (3)	−0.0089 (3)
O2	0.0406 (13)	0.0265 (11)	0.0367 (13)	−0.0077 (10)	−0.0072 (10)	−0.0060 (9)
O3	0.0534 (15)	0.0261 (12)	0.0377 (13)	−0.0049 (11)	−0.0148 (12)	−0.0006 (10)
O4	0.0473 (14)	0.0244 (11)	0.0421 (14)	−0.0053 (10)	−0.0126 (11)	−0.0047 (10)
O5	0.0500 (15)	0.0329 (14)	0.0455 (15)	−0.0011 (11)	−0.0215 (12)	−0.0075 (11)
N2	0.0313 (13)	0.0201 (12)	0.0264 (13)	−0.0064 (10)	−0.0015 (10)	−0.0034 (10)
C10	0.0319 (16)	0.0233 (15)	0.0291 (16)	−0.0063 (12)	−0.0023 (12)	−0.0045 (12)
C11	0.0397 (18)	0.0345 (17)	0.0343 (18)	−0.0105 (15)	−0.0031 (14)	−0.0115 (14)
C12	0.0421 (19)	0.0235 (15)	0.045 (2)	−0.0070 (14)	−0.0021 (15)	−0.0141 (14)
C13	0.0365 (17)	0.0200 (14)	0.0412 (18)	−0.0038 (13)	0.0000 (14)	−0.0068 (13)
C14	0.0320 (15)	0.0202 (14)	0.0278 (15)	−0.0040 (12)	0.0002 (12)	−0.0029 (11)
C15	0.0323 (16)	0.0237 (15)	0.0288 (16)	−0.0046 (12)	0.0008 (13)	−0.0033 (12)
C16	0.0354 (17)	0.0300 (16)	0.0289 (16)	−0.0070 (13)	−0.0027 (13)	−0.0058 (12)
O1	0.0432 (15)	0.0338 (13)	0.0582 (17)	−0.0056 (11)	−0.0071 (13)	−0.0038 (12)
N1	0.0370 (15)	0.0378 (16)	0.0420 (17)	−0.0127 (13)	0.0063 (13)	−0.0134 (13)
C1	0.0340 (17)	0.0346 (18)	0.0403 (19)	−0.0080 (14)	0.0051 (14)	−0.0050 (14)
C2	0.0392 (19)	0.050 (2)	0.041 (2)	−0.0050 (17)	−0.0003 (16)	−0.0087 (16)
C3	0.050 (2)	0.057 (2)	0.052 (2)	−0.0187 (19)	0.0040 (18)	−0.0258 (19)
C4	0.053 (2)	0.037 (2)	0.059 (2)	−0.0130 (17)	0.0070 (19)	−0.0170 (17)
C5	0.045 (2)	0.0368 (19)	0.052 (2)	−0.0038 (16)	0.0085 (17)	−0.0038 (17)
C6	0.043 (2)	0.055 (2)	0.047 (2)	−0.0026 (19)	−0.0058 (18)	0.0016 (19)
C7	0.0388 (19)	0.061 (2)	0.041 (2)	−0.0171 (18)	0.0012 (16)	−0.0131 (18)
C8	0.0289 (16)	0.0347 (17)	0.0376 (18)	−0.0067 (13)	0.0079 (13)	−0.0080 (14)
C9	0.0407 (19)	0.0330 (17)	0.0414 (19)	−0.0073 (15)	0.0057 (15)	−0.0076 (14)
O1W	0.0545 (18)	0.0310 (14)	0.0588 (18)	−0.0029 (13)	−0.0052 (15)	−0.0001 (13)
O2W	0.0608 (19)	0.0482 (17)	0.060 (2)	−0.0136 (15)	0.0024 (16)	−0.0059 (15)

### 8-Hydroxyquinolinium trichlorido(pyridine-2,6-dicarboxylic acid-κ3O,N,O')copper(II) dihydrate Geometric parameters (Å, º)

**Table:**

Cu1—Cl1	2.3688 (11)	O1—C1	1.357 (4)
Cu1—Cl2	2.2067 (9)	N1—H1A	0.863 (10)
Cu1—Cl3	2.4190 (10)	N1—C7	1.323 (5)
Cu1—O2	2.424 (2)	N1—C8	1.369 (4)
Cu1—O4	2.366 (2)	C1—C2	1.365 (5)
Cu1—N2	2.011 (2)	C1—C8	1.407 (5)
O2—C15	1.205 (4)	C2—H2	0.9300
O3—H3	0.822 (10)	C2—C3	1.414 (6)
O3—C15	1.312 (4)	C3—H3A	0.9300
O4—C16	1.212 (4)	C3—C4	1.359 (6)
O5—H5	0.818 (10)	C4—H4	0.9300
O5—C16	1.295 (4)	C4—C9	1.402 (5)
N2—C10	1.343 (4)	C5—H5A	0.9300
N2—C14	1.338 (4)	C5—C6	1.360 (6)
C10—C11	1.377 (4)	C5—C9	1.405 (5)
C10—C16	1.508 (4)	C6—H6	0.9300
C11—H11	0.9300	C6—C7	1.384 (6)
C11—C12	1.386 (5)	C7—H7	0.9300
C12—H12	0.9300	C8—C9	1.417 (5)
C12—C13	1.377 (5)	O1W—H1WA	0.847 (10)
C13—H13	0.9300	O1W—H1WB	0.844 (10)
C13—C14	1.382 (4)	O2W—H2WA	0.853 (10)
C14—C15	1.506 (4)	O2W—H2WB	0.850 (10)
O1—H1	0.822 (10)		
			
Cl1—Cu1—Cl3	174.14 (3)	O2—C15—C14	121.7 (3)
Cl1—Cu1—O2	90.47 (6)	O3—C15—C14	112.2 (3)
Cl2—Cu1—Cl1	93.30 (4)	O4—C16—O5	126.2 (3)
Cl2—Cu1—Cl3	92.50 (4)	O4—C16—C10	120.6 (3)
Cl2—Cu1—O2	104.88 (6)	O5—C16—C10	113.1 (3)
Cl2—Cu1—O4	105.45 (6)	C1—O1—H1	110 (3)
Cl3—Cu1—O2	87.19 (6)	C7—N1—H1A	119 (3)
O4—Cu1—Cl1	92.42 (7)	C7—N1—C8	122.6 (3)
O4—Cu1—Cl3	86.88 (7)	C8—N1—H1A	118 (3)
O4—Cu1—O2	149.30 (8)	O1—C1—C2	125.7 (3)
N2—Cu1—Cl1	86.29 (8)	O1—C1—C8	115.5 (3)
N2—Cu1—Cl2	179.23 (8)	C2—C1—C8	118.8 (3)
N2—Cu1—Cl3	87.90 (8)	C1—C2—H2	119.8
N2—Cu1—O2	74.48 (9)	C1—C2—C3	120.4 (4)
N2—Cu1—O4	75.23 (9)	C3—C2—H2	119.8
C15—O2—Cu1	107.79 (19)	C2—C3—H3A	119.3
C15—O3—H3	108 (3)	C4—C3—C2	121.3 (4)
C16—O4—Cu1	109.41 (19)	C4—C3—H3A	119.3
C16—O5—H5	106 (4)	C3—C4—H4	120.1
C10—N2—Cu1	119.7 (2)	C3—C4—C9	119.9 (4)
C14—N2—Cu1	121.0 (2)	C9—C4—H4	120.1
C14—N2—C10	119.0 (3)	C6—C5—H5A	119.3
N2—C10—C11	122.0 (3)	C6—C5—C9	121.4 (4)
N2—C10—C16	114.3 (3)	C9—C5—H5A	119.3
C11—C10—C16	123.7 (3)	C5—C6—H6	120.4
C10—C11—H11	120.5	C5—C6—C7	119.2 (4)
C10—C11—C12	119.1 (3)	C7—C6—H6	120.4
C12—C11—H11	120.5	N1—C7—C6	120.6 (4)
C11—C12—H12	120.6	N1—C7—H7	119.7
C13—C12—C11	118.7 (3)	C6—C7—H7	119.7
C13—C12—H12	120.6	N1—C8—C1	120.3 (3)
C12—C13—H13	120.3	N1—C8—C9	118.8 (3)
C12—C13—C14	119.4 (3)	C1—C8—C9	120.9 (3)
C14—C13—H13	120.3	C4—C9—C5	124.0 (3)
N2—C14—C13	121.8 (3)	C4—C9—C8	118.6 (3)
N2—C14—C15	114.2 (3)	C5—C9—C8	117.3 (3)
C13—C14—C15	124.0 (3)	H1WA—O1W—H1WB	100 (5)
O2—C15—O3	126.0 (3)	H2WA—O2W—H2WB	114 (6)
			
Cu1—O2—C15—O3	171.8 (3)	C14—N2—C10—C16	−176.3 (3)
Cu1—O2—C15—C14	−7.7 (4)	C16—C10—C11—C12	176.5 (3)
Cu1—O4—C16—O5	179.5 (3)	O1—C1—C2—C3	−178.4 (3)
Cu1—O4—C16—C10	−1.1 (4)	O1—C1—C8—N1	0.1 (5)
Cu1—N2—C10—C11	−172.8 (3)	O1—C1—C8—C9	−179.9 (3)
Cu1—N2—C10—C16	9.7 (4)	N1—C8—C9—C4	177.9 (3)
Cu1—N2—C14—C13	173.5 (2)	N1—C8—C9—C5	−0.3 (5)
Cu1—N2—C14—C15	−8.1 (4)	C1—C2—C3—C4	−0.8 (6)
N2—C10—C11—C12	−0.8 (5)	C1—C8—C9—C4	−2.1 (5)
N2—C10—C16—O4	−5.1 (5)	C1—C8—C9—C5	179.7 (3)
N2—C10—C16—O5	174.4 (3)	C2—C1—C8—N1	−178.1 (3)
N2—C14—C15—O2	11.1 (5)	C2—C1—C8—C9	1.9 (5)
N2—C14—C15—O3	−168.5 (3)	C2—C3—C4—C9	0.7 (6)
C10—N2—C14—C13	−0.4 (5)	C3—C4—C9—C5	178.9 (4)
C10—N2—C14—C15	178.0 (3)	C3—C4—C9—C8	0.8 (6)
C10—C11—C12—C13	−0.4 (5)	C5—C6—C7—N1	−1.9 (6)
C11—C10—C16—O4	177.5 (3)	C6—C5—C9—C4	−179.1 (4)
C11—C10—C16—O5	−3.1 (5)	C6—C5—C9—C8	−0.9 (6)
C11—C12—C13—C14	1.2 (5)	C7—N1—C8—C1	−179.5 (3)
C12—C13—C14—N2	−0.8 (5)	C7—N1—C8—C9	0.5 (5)
C12—C13—C14—C15	−179.1 (3)	C8—N1—C7—C6	0.7 (6)
C13—C14—C15—O2	−170.6 (3)	C8—C1—C2—C3	−0.5 (6)
C13—C14—C15—O3	9.8 (5)	C9—C5—C6—C7	2.1 (6)
C14—N2—C10—C11	1.2 (5)		

### 8-Hydroxyquinolinium trichlorido(pyridine-2,6-dicarboxylic acid-κ3O,N,O')copper(II) dihydrate Hydrogen-bond geometry (Å, º)

**Table:**

*D*—H···*A*	*D*—H	H···*A*	*D*···*A*	*D*—H···*A*
O1—H1···Cl1	0.82 (4)	2.31 (4)	3.124 (3)	172 (3)
O1*W*—H1*WA*···O2*W*	0.85 (4)	1.86 (4)	2.697 (5)	172 (5)
N1—H1*A*···Cl3^i^	0.86 (2)	2.41 (3)	3.201 (3)	154 (5)
O1*W*—H1*WB*···O4^ii^	0.84 (4)	2.04 (3)	2.808 (4)	152 (4)
O3—H3···Cl3^iii^	0.82 (3)	2.20 (3)	3.015 (3)	173 (4)
O2*W*—H2*WA*···Cl2^iv^	0.86 (6)	2.53 (5)	3.361 (4)	163 (5)
O2*W*—H2*WB*···Cl1^ii^	0.85 (5)	2.50 (5)	3.317 (4)	160 (4)
O5—H5···O1*W*	0.82 (4)	1.69 (4)	2.477 (4)	162 (5)
C3—H3*A*···O2*W*^v^	0.93	2.56	3.473 (5)	168
C6—H6···Cl2^vi^	0.93	2.78	3.622 (4)	151
C7—H7···O2^vi^	0.93	2.44	3.356 (4)	169
C11—H11···O5^vii^	0.93	2.56	3.396 (5)	150
C12—H12···O1*W*^vii^	0.93	2.58	3.412 (5)	148

Symmetry codes: (i) *x*−1, *y*, *z*; (ii) −*x*+2, −*y*, −*z*+1; (iii) −*x*+1, −*y*+1, −*z*; (iv) *x*+1, *y*, *z*; (v) *x*−1, *y*+1, *z*; (vi) −*x*, −*y*+1, −*z*; (vii) −*x*+2, −*y*+1, −*z*+1.
